# Investigating Possible Interspecies Communication of Plasmids Associated with Transfer of Third-Generation Cephalosporin, Quinolone, and Colistin Resistance Between Simultaneously Isolated Escherichia Coli and Klebsiella Pneumoniae

**DOI:** 10.1128/spectrum.03554-22

**Published:** 2023-05-01

**Authors:** Jingjing Quan, Huangdu Hu, Huichuan Zhang, Yan Meng, Weichao Liao, Junxin Zhou, Xinhong Han, Qiucheng Shi, Dongdong Zhao, Qian Wang, Yan Jiang, Yunsong Yu

**Affiliations:** a Department of Infectious Diseases, Sir Run Run Shaw Hospital, Zhejiang University School of Medicine, Hangzhou, Zhejiang, China; b Key Laboratory of Microbial Technology and Bioinformatics of Zhejiang Province, Hangzhou, Zhejiang, China; c Regional Medical Center for National Institute of Respiratory Diseases, Sir Run Run Shaw Hospital, Zhejiang University School of Medicine, Hangzhou, Zhejiang, China; d Ningbo Institute of Innovation for Combined Medicine and Engineering; e Department of Infectious Diseases, Ningbo Medical Center Lihuili Hospital, Ningbo, Zhejiang, China; f Department of Clinical Laboratory, Zhejiang Hospital, Zhejiang University School of Medicine, Hangzhou, Zhejiang, China; g Department of Intensive Care Unit, Sir Run Run Shaw Hospital, Zhejiang University School of Medicine, Hangzhou, Zhejiang, China; h Department of General Practice, Sir Run Run Shaw Hospital, Zhejiang University School of Medicine, Hangzhou, Zhejiang, China; Tianjin University

**Keywords:** interspecies, plasmid, colistin, *mcr-1*, IS*26*

## Abstract

The coinfection process producing multiple species of pathogens provides a specific ecological niche for the exchange of genetic materials between pathogens, in which plasmids play a vital role in horizontal gene transfer, especially for drug resistance, but the underlying transfer pathway remains unclear. Interspecies communication of the plasmids associated with the transfer of third-generation cephalosporins, quinolones, and colistin resistance has been observed in simultaneously isolated Escherichia coli and Klebsiella pneumoniae from abdominal drainage following surgery. The MICs of antimicrobial agents were determined by the broth microdilution method. The complete chromosome and plasmid sequences were obtained by combining Illumina paired-end short reads and MinION long reads. S1-PFGE, southern blot analysis and conjugation assay confirmed the transferability of the *mcr-1*-harboring plasmid. Both the E. coli isolate EC15255 and K. pneumoniae isolate KP15255 from the same specimen presented multidrug resistance. Each of them harbored one chromosome and three plasmids, and two plasmids and their mediated resistance could be transferred to the recipient by conjugation. Comparison of their genome sequences suggested that several genetic communication events occurred between species, especially among their plasmids, such as whole-plasmid transfer, insertion, deletion, amplification, or inversion. Exchange of plasmids or the genetic elements they harbor plays a critical role in antimicrobial resistance gene transmission and poses a substantial threat to nosocomial infection control, necessitating the continued surveillance of multidrug resistant pathogens, especially during coinfection.

**IMPORTANCE** The genome sequence of bacterial pathogens commonly provides a detailed clue of genetic communication among clones or even distinct species. The intestinal microecological environment is a representative ecological niche for genetic communication. However, it is still difficult to describe the details of horizontal gene transfer or other genetic events within them because the evidence in the genome sequence is incomplete and limited. In this study, the simultaneously isolated Escherichia coli and Klebsiella pneumoniae from a coinfection process provided an excellent example for observation of interspecies communication between the two genomes and the plasmids they harbor. A complete genome sequence acquired by combining the Illumina and MinION sequencing platforms facilitated the understanding of genetic communication events, such as whole-plasmid transfer, insertion, deletion, amplification, or inversion, which contribute to antimicrobial resistance gene transmission and are a substantial threat to nosocomial infection control.

## INTRODUCTION

*Enterobacteriales* are normally opportunistic pathogens that cause severe nosocomial infections, including pneumonia, bloodstream, and abdominal infections, and have been of more clinical concern owing to their association with multidrug resistance spreading worldwide (1, 2). Escherichia coli and Klebsiella pneumoniae are the two most epidemic bacterial pathogens belonging to *Enterobacteriales* that cause nosocomial infections, with isolation rates of approximately 18.96% and 14.12%, respectively, in China in 2021 (data from China Antimicrobial Surveillance Network, CHINET). Extended-spectrum β-lactamases (ESBLs) are very popular in clinically isolated E. coli or K. pneumoniae, resulting in resistance against most β-lactam antibiotics, even third-generation cephalosporins (3, 4). Some aminoglycoside-modifying enzymes, such as AACs, APHs, ANTs, etc., and the pentapeptide repeat protein QNR family mediate resistance to aminoglycosides and quinolones, respectively (5–7). Furthermore, the last-resort antibiotic, colistin, for treating multidrug resistant bacterial infections, even faced the challenges of resistance mediated by the *mcr-1* gene and its homologs (8, 9). All of the resistance determinants mentioned above are commonly encoded by plasmids, which are the most dynamic vehicles for genetic material exchange, accelerating the dissemination of antimicrobial agent resistance (10–13).

Coinfection that often occurs clinically implies that multiple species of pathogens probably emerge in the same anatomic site, providing an ecological niche for the exchange of genetic materials among the mixed pathogens (14). That is also a specific evolution event for microbes occurring within the host during the infection process, among which horizontal gene transfer is most frequent, especially between the bacteria from *Enterobacteriales* (14). Plasmids play a vital role in horizontal gene transfer, especially for antimicrobial resistance determinants, because many acquired antimicrobial resistance genes are mobilized by plasmid conjugation or by mobile elements located on plasmids, such as insertion sequences (ISs), transposons or integrons (10, 13, 15).

Although numerous studies have reported the horizontal transfer of antimicrobial resistance genes in *Enterobacteriales*, detailed observation of the genetic exchange between distinct species of *Enterobacteriales* within host coinfection is still limited. It is difficult to clarify the border of mobile elements or the whole transferred segment because the draft of the bacterial genome we normally acquire based on the Illumina short-read sequencing platform is fragmented (16). Nevertheless, assembly combined with long-read sequences, such as the Nanopore or PacBio platform, and Illumina short-read sequences could solve this major problem (17, 18). Hence, in this study, we used this sequencing strategy to investigate the exchange of plasmid or plasmid-harboring mobile elements with resistance genes, including the transmission of resistance genes against third-generation cephalosporins, quinolones and colistin, between E. coli and K. pneumoniae isolated from a coinfection patient.

## RESULTS

### Isolate characteristics.

Both the E. coli isolate EC15255 and K. pneumoniae isolate KP15255 were isolated simultaneously from abdominal drainage collected following surgery of a patient and presented multidrug resistance against colistin, cefepime, cefoperazone/sulbactam, levofloxacin, aztreonam, tetracycline, and sulfamethoxazole (Table 1). The MIC of colistin, the last resort antimicrobial agent for Gram-negative severe infection, reached 4 mg/L and 16–32 mg/L, respectively.

**TABLE 1:** MICs of antimicrobial agents against the E. coli and K. pneumoniae clinical isolates and their transconjugants

Strains	CST^*d*^	CAZ	FEP	CPS	PTC	ETP	MEM
	MIC (mg/L)						
EC15255	**4** ^*e*^	8–16	**256**	**64–128**	32	0.06–0.25	0.015–0.06
EC15255C^*a*^	**4**	8–32	**64–128**	**64–128**	16–32	0.125–0.25	0.03–0.125
KP15255	**16–32**	8–16	**64**	**128–256**	32	0.125–0.5	0.03–0.06
KP15255C^*b*^	**4**	0.5	0.06–0.125	0.25–0.5	2–4	0.008–0.015	0.03–0.06
EC600	0.03–0.06	0.25–0.5	0.06–0.125	0.25–0.5	4	0.008–0.03	0.03–0.06
ATCC 25922^*c*^	0.25	0.125–0.25	0.03–0.125	0.125–0.25	2	0.008–0.03	0.015–0.06
	**ATM**	**LEV**	**AMK**	**TGC**	**MIN**	**TET**	**SUL**
**Strains**	**MIC (mg/L)**						
EC15255	**16–32**	**8–16**	4–16	0.25	4	**128**	**>1024**
EC15255C^*a*^	**32–64**	**4–8**	4–8	0.060.125	1–2	0.25–0.5	**1024**
KP15255	**16–32**	**16**	16–32	0.5–1	**128**	**>256**	**>1024**
KP15255C^*b*^	0.125–0.25	**4–8**	32–64	0.125	1	0.25–0.5	**1024**
EC600	0.125–0.25	0.125	4–8	0.125–0.5	0.5–1	0.25–0.5	**512–1024**
ATCC 25922^*c*^	0.03–0.06	0.008–0.03	4	0.06	0.125–0.25	0.25–0.5	16–32

a Transconjugant of E. coli isolate EC15255.

b Transconjugant of K. pneumoniae isolate KP15255.

c Quality control for antimicrobial susceptibility testing.

d CST, colistin; CAZ, ceftazidime; FEP, cefepime; CPS, cefoperazone/sulbactam; PTC, piperacillin-tazobactam; ETP, ertapenem; MEM, meropenem; ATM, aztreonam; LEV, levofloxacin; AMK, amikacin; TGC, tigecycline; MIN, minocycline; TET, tetracycline; SMZ, sulfamethoxazole.

e Bold text indicates resistance.

### Conjugation assay.

**FIG 1:**
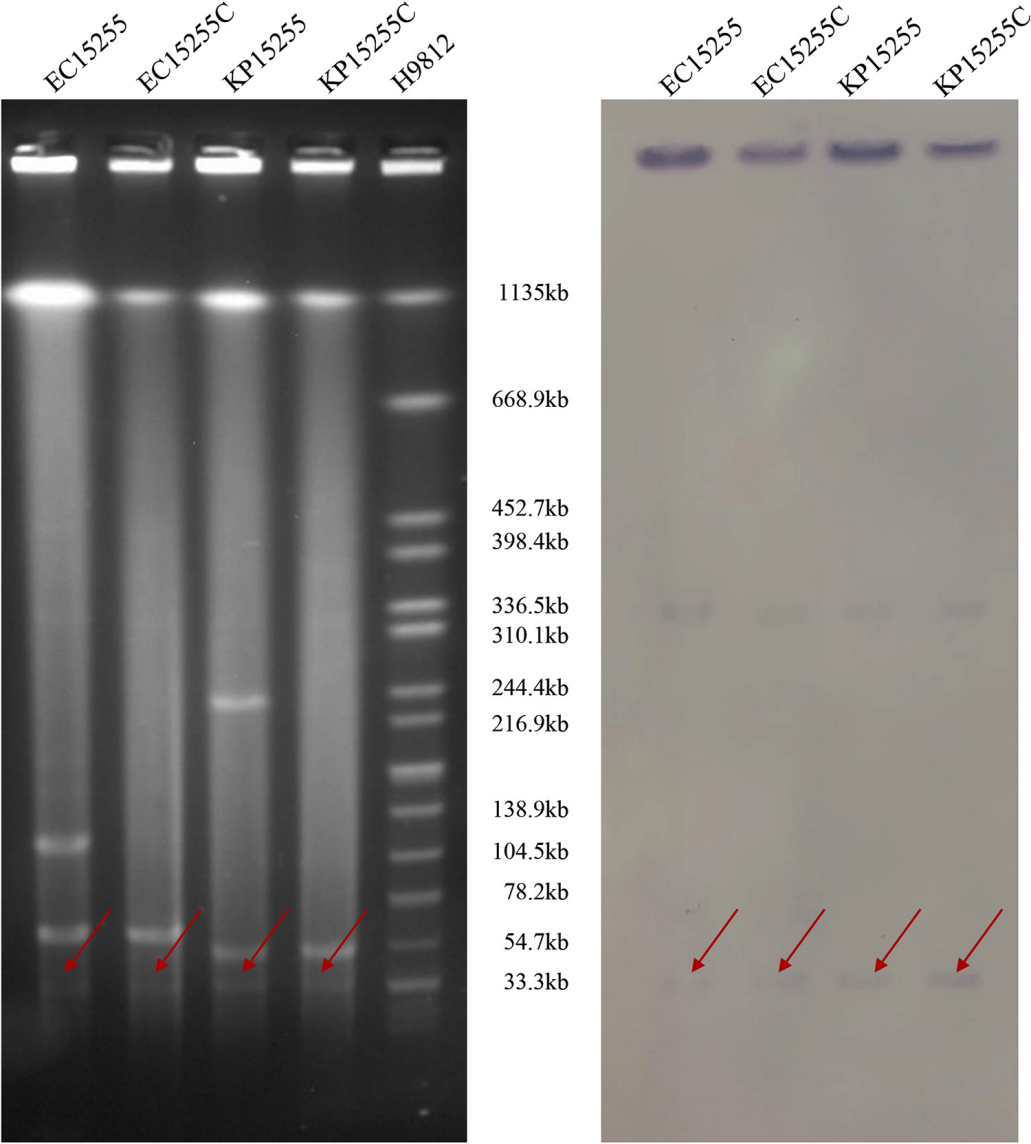
S1-digested plasmid DNA and Southern blot hybridization with the *mcr-1* gene of E. coli isolate EC15255, K. pneumoniae isolate KP15255, and their transconjugants. The red arrows indicate positive signals via southern blot hybridization with the *mcr-1* probes.

Conjugation assays confirmed that colistin resistance could be transferred from both donor strains to the recipient strain EC600. The conjugation efficiencies showed that the colistin resistance could be successfully transferred from E. coli EC15255 to E. coli EC600 at a frequency of (2.33 to 6.49)*10^–^³ cells per recipient cell, while for K. pneumoniae KP15255, the frequency was (1.59 to 2.23)*10^−4^ (*P* = 0.0582) (shown in Fig. S1). PCR screening of *mcr* family genes and subsequent sequencing revealed that they carried *mcr-1*. Further S1-PFGE and Southern blot analysis determined that the two *mcr-1* genes were located on plasmids of the same size, which was approximately 33 kb (Fig. 1). The MICs of colistin for their transconjugants both increased from 0.03–0.06 mg/L to 4 mg/L (Table 1). Interestingly, the antimicrobial resistance that could be cotransferred to the recipient strain presented distinct results; for instance, quinolone resistance could be cotransferred from both but β-lactam only for E. coli and aminoglycoside (the MIC of amikacin increased significantly but did not present resistance) only for K. pneumoniae.

### Analysis of the WGS.

To determine the resistance determinants and the potential genetic communication between the two strains that were isolated from the same specimen, their complete genome sequences were obtained by combining Illumina short-read and MinION long-read sequencing. Genome analyses revealed that both the E. coli and K. pneumoniae isolates contained one complete chromosome and three circular plasmids, harboring dozens of acquired resistance genes. All three plasmids of EC15255 and KP15255 harbored mobile-associated genes, such as *tra*, *trb*, and *trw*. Detailed information is listed in the supplemental materials (Table S1).

E. coli isolate EC15255 belonged to ST226. Its chromosome was 4.63 Mbp in size and carried resistance genes such as *aac*(3)*-Iid*, *aph(3″)-Ib*, *aph*(6)*-Id*, *bla*_TEM-1B,_ *mdf(A)*, *mpf(A)*, *catA1*, *sul2*, and *tet(A)*. A relatively “large” plasmid pEC15255_1 with 119.5 Kbp in size carried *aac(6′)-Ib3*, *aac(6′)-Ib-cr*, *aph(3″)-Ib*, *aph*(6)*-Id*, *cmlA1*, *sul2*, *tet(A)*, *bla*_CTX-M-14_, and *bla*_TEM-1B_. The next “middle” plasmid pEC15255_2 carried *bla*_CTX-M-14_, two copies of *qnrS1*, and *dfrA14* with 65.8 Kbp in size, while the last “small” plasmid pEC15255_3 only carried the *mcr-1.1* gene with 33.3 Kbp in size. Note that *aph(3″)-Ib*, *aph*(6)*-Id*, *qnrS1*, *sul2*, *tet(A)*, *bla*_TEM-1B_, and *bla*_CTX-M-14_ all had two copies (Table 2).

**TABLE 2:** Resistance genes located in the genomes of E. coli and K. pneumoniae isolates and Inc types of plasmids

Isolates	ST	Chromosome (bp)	Plasmid 1 (bp)	Plasmid 2 (bp)	Plasmid 3 (bp)
EC15255	226	4,634,848	119,528	65,802	33,309
Antimicrobials resistance gene		*aac*(3)*-Iid, aph(3″)-Ib, aph*(6)*-Id, bla*_TEM−1B_, *mdf(A), mpf(A), catA1, sul2, tet(A)*	*aac(6′)-Ib3, aac(6′)-Ib-cr, aph(3″)-Ib, aph*(6)*-Id, bla*_CTX-M-14_*, bla*_TEM-1B_*, cmlA1, sul2, tet(A)*	*bla*_CTX-M-14_, *qnrS1, qnrS1, dfrA14*	*mcr-1.1*
Inc types		/	IncFIB/FII/Q1	IncN/U	IncX4
KP15255	2570	5,129,789	324,101	56,347	33,309
Antimicrobials resistance gene		*bla*_SHV-81_, *oqxA, oqxB*	*aac*(3)*-Iid, bla*_CTX-M-14_*, bla*_CTX-M-14_*, bla*_LAP-2_*, catA2, qnrS1, tet(D)*	*aac(6′)-Ib-cr, qnrS1, ARR-3, dfrA14*	*mcr-1.1*
Inc types		/	IncFIB/FII	IncN/U	IncX4

K. pneumoniae isolate KP15255 belonged to ST2570. Similar to E. coli EC15255, KP15255 also carried a variety of antimicrobial resistance genes, among which *bla*_SHV-81,_ *oqxA*, and *oqxB* were carried by the chromosome; *aac*(3)*-Iid*, two copies of *bla*_CTX-M-14_, *bla*_LAP-2_, *catA2*, *qnrS1*, and *tet(D)* were carried by the “large” plasmid pKP15255_1 that was 324.1 Kbp in size; *aph(3″)-Ib*, *aac(6′)-Ib-cr*, *ARR-3*, and *dfrA14* were carried by the “middle” plasmid pKP15255_2 that was 56.3 Kbp in size; and the *mcr-1.1* gene was located on the “small” plasmid pKP15255_3, which was the same size as the *mcr-1*-harboring plasmid pEC15255_3 in E. coli. The genes *qnrS1* and *bla*_CTX-M-14_ had two copies (Table 2).

### Genetic communication events among plasmids.

**FIG 2:**
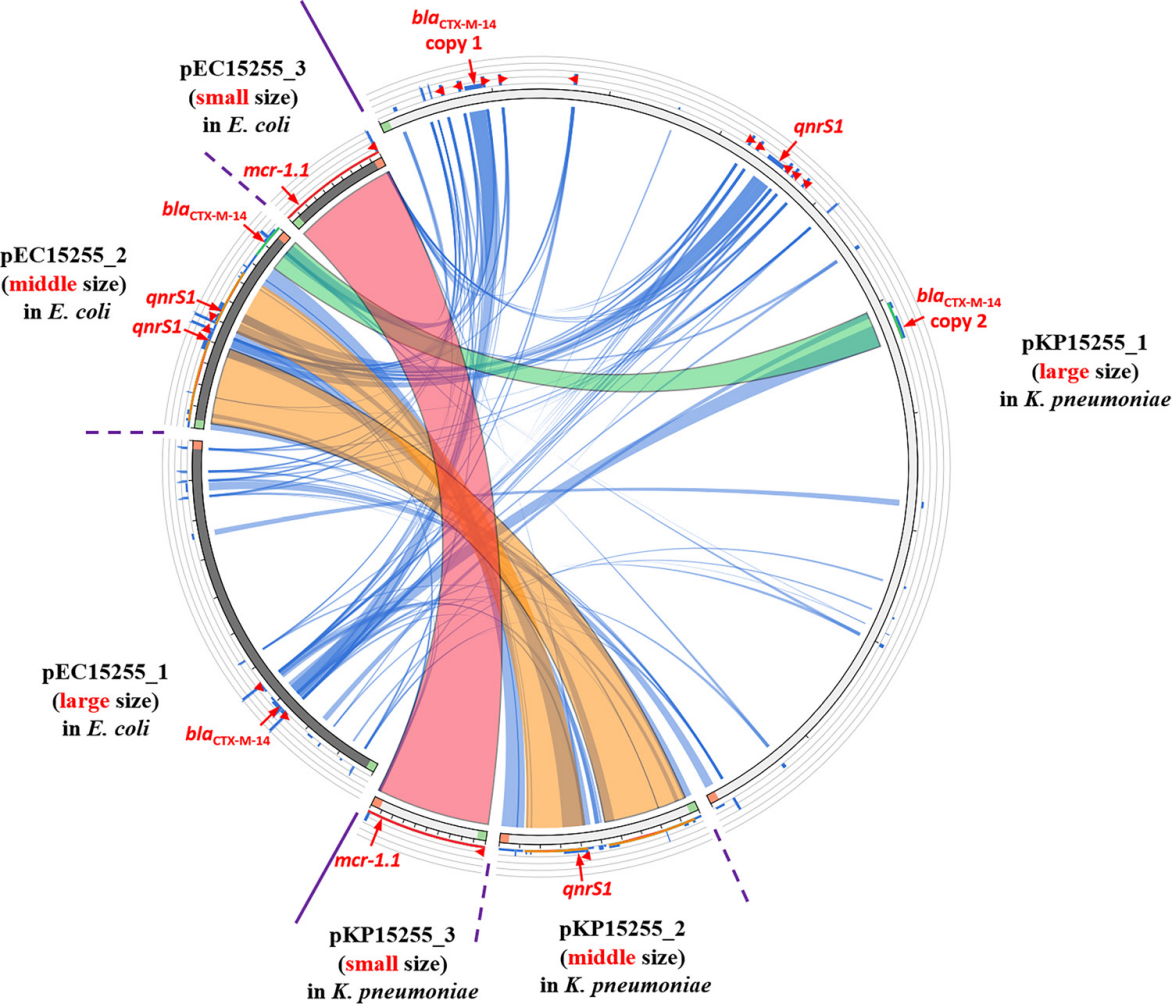
The schematic relationship among plasmids in E. coli and K. pneumoniae based on sequence similarities was visualized by Circos. For the circle, three arc segments from 7 to 11 o'clock clockwise (left part of the circle) represent the large, middle, and small plasmids in E. coli, respectively. Similarly, another three arc segments at the right part of the circle represent the three plasmids in K. pneumoniae. The ESBL gene *bla*_CTX-M-14_, quinolone resistance gene *qnrS1* and colistin resistance gene *mcr-1.1* are marked with their relative position on the plasmid by small red arrows. The small triangles on the plasmid indicate the IS*26* elements at multiple sites.

Complete genome sequence analysis suggested that several genetic communication events occurred between the two species, especially among their plasmids, such as whole-plasmid transfer, insertion/deletion (in/del), amplification, or inversion. Both *mcr-1*-harboring plasmids in E. coli and K. pneumoniae isolates were completely identical (100%), which could imply the whole-plasmid interchange between two isolates collected from the same specimen. This IncX4-type plasmid was 33,309 bp in length with an average GC content of 41.8%. The genetic environment of the *mcr-1* gene in this IncX4 plasmid is an incomplete Tn*6330* structure, in which the conserved *pap2* was located downstream of the *mcr-1.1* gene and two copies of IS*Apl1* that commonly transfer the *mcr-1.1* gene were not found, indicating that the transfer of *mcr-1.1* was mediated by plasmid mobility rather than small mobile segments. Even a few mobile segments, such as IS or transposons, except for a sole IS*26* segment, were found in this plasmid, indicating its conserved genetic structure (Fig. 2).

**FIG 3:**
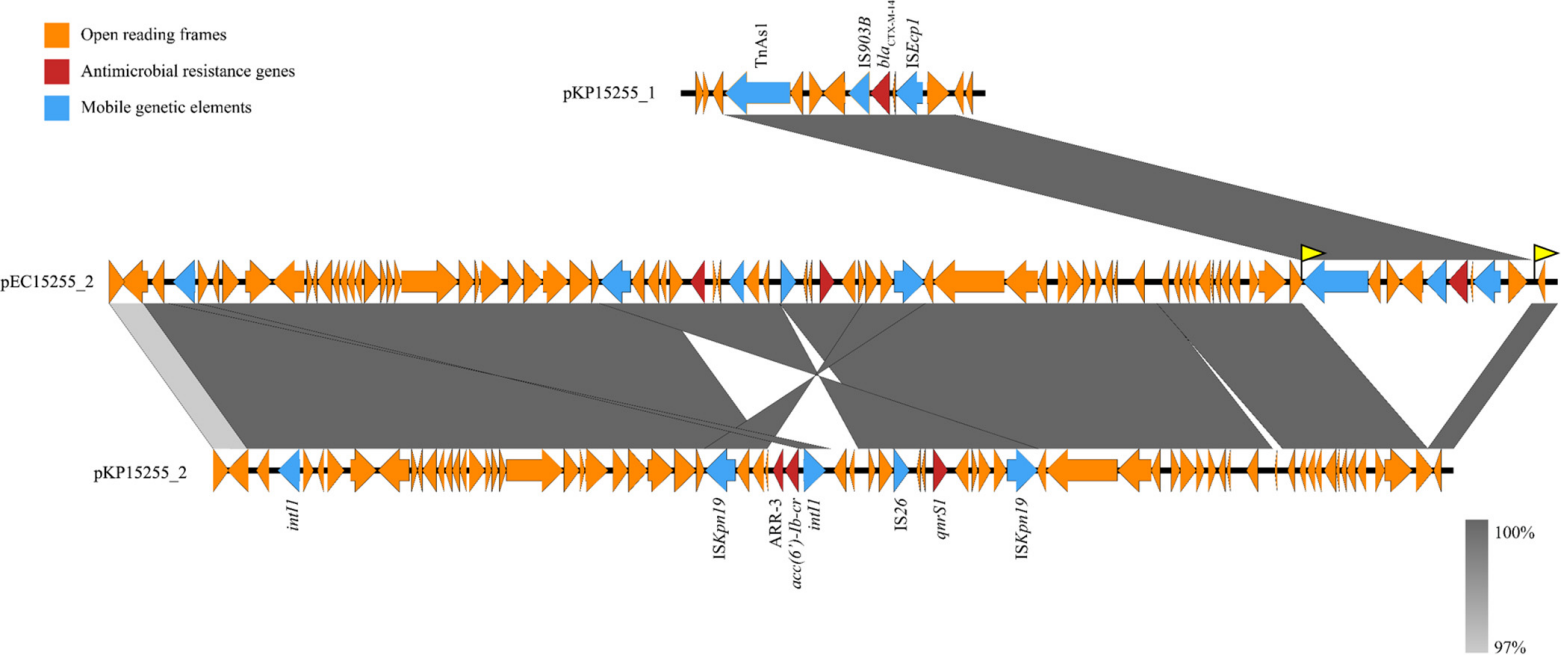
Comparison of the Tn*As1*-IS*903B*-*bla*_CTX-M-14_-IS*Ec9* cassette between the plasmid pEC15255_2 and pKP15255_2 backgrounds. The yellow flag represents the direct repeats (DRs) (CTATATTG).

The results indicated that multiple events might have occurred in the sequence of the middle-sized plasmid in each isolate. First, both plasmids (pEC15255_2 and pKP15255_2) were IncN/U type, and their backbones were similar and shared 91.5% identity (based on pKP15255_2). We speculated that plasmid interchange might be occurred between the two isolates. Second, compared with the middle plasmid pKP15255_2, the middle plasmid in E. coli (pEC15255_2) has been inserted with a 10,441-bp fragment, which harbored the cassette of Tn*As1*-IS*903B*-*bla*_CTX-M-14_-IS*Ecp1*. Transposon Tn*As1*, rather than IS*Ecp1* or IS*903B*, mediated the insertion event because the 32-bp inverted sequences (IRs) of Tn*As1* and 8-bp direct sequences (DRs) have been found to seamlessly flank the insertion fragment (Fig. 3). Interestingly, it seemed that this 10,441-bp region was from the plasmid pKP15255_1, the largest plasmid in K. pneumoniae. Finally, an inversion event and multiple copies occurred between the two middle plasmids. An 8181-bp *qnrS1*-harboring fragment in pEC15255_2 of E. coli was amplified and inserted inversely, resulting in a concurrent 5705-bp deletion during the process compared to pKP15255_2 of K. pneumoniae (Fig. 3).

The large plasmids in each species are substantially different. However, both of them harbored *bla*_CTX-M-14_ genes, one in a large plasmid of E. coli and two copies in that of K. pneumoniae. Copy 1 of *bla*_CTX-M-14_ is located on the plasmid pKP15255_1 with the structure IS*903B*-*bla*_CTX-M-14_-IS*Ecp1* and was identified with that in pEC15255_1 but lacked the Tn*As1* transposonase gene compared to *bla*_CTX-M-14_ copy 2. That was the reason why we speculated that *bla*_CTX-M-14_ copy 2 was inserted into the middle plasmid pEC15255_2 in E. coli, rather than *bla*_CTX-M-14_ copy 1. The latter was associated with another copy of *bla*_CTX-M-14_ in the large plasmid pEC15255_1 in E. coli, possibly mediated by the flanked IS*26* elements they both harbored (Fig. 2).

## DISCUSSION

Bacterial coinfection complicates the choice of clinical therapy for patients and facilitates the dissemination of antimicrobial resistance through horizontal gene transfer when at least one bacterial pathogen in the coinfection environment harbors mobile resistant determinants (14). Here, we reported a coinfection caused by a mix of E. coli and K. pneumoniae and observed genetic communication among their harbored plasmids and other mobile elements based on complete genome sequence analysis. Although the genome sequence provided a sketch for possible genetic communication between distinct species and its evolutionary direction was still unclear, we confirmed that exchange events have occurred and exhibited the plasticity of plasmids that presented multidrug resistance.

Plasticity of plasmids is an important feature in resistant bacterial evolution (15). Plasmids are considered not only the key vector for genetic exchange but also an important contributor to the novelty and evolution of prokaryotic genomes (19). In this study, we investigated the interchange of plasmids or plasmid-harboring mobile elements with resistance genes between E. coli and K. pneumoniae isolated from the same patient. There are three plasmids harbored by E. coli EC15255 and K. pneumoniae KP15255 strains, all of which present multiple resistance, encoding a series of aminoglycoside modifying enzymes, β-lactams, pentapeptide repeat protein QNR family, etc. The plasmid types of the two isolates included IncFIB/FII/Q1, IncFIB/FII, IncN/U and IncX4. Except for the IncX4, the other three plasmid types were all multireplicons. All the four plasmid types were common in *Enterobacteriales*, especially in E. coli and K. pneumoniae isolates. For the IncFIB/FII/Q1-type plasmid, it could be harbored by E. coli isolates collected from patients with urinary tract infections (20). As for the IncFIB/FII-type plasmid, it was usually harbored by K. pneumoniae isolates, which were isolated from sputum, infected chronic wounds, or human feces (21–23). For the IncN/U-type plasmid, it could also be harbored by E. coli and K. pneumoniae isolates, which were isolated from sputum, urine, etc. (genome accession no. CP072981, CP091849, CP092655, etc.). The IncX4-type is one of the most common plasmid types of *mcr-1*-carrying plasmids, which could be found in E. coli, K. pneumoniae and Salmonella enterica isolates collected from poultry, human or environment (24).

In the present study, the two isolates were isolated from abdominal drainage specimen of a patient who had suffered intestinal perforation and underwent surgical treatment. So we speculated that the two pathogens were more probably originated from the intestinal tract, where E. coli and K. pneumoniae normally colonized, rather than the environment. However, it was indeed a pity that we could not confirm E. coli was the parental isolate that carried these two plasmids and transferred them to K. pneumoniae or *vice versa*.

The ability of plasmid conjugation was confirmed by a filter mating test and sequence analysis. Based on the plasmid sequence annotation, the conjugation-related gene cluster was found to be distributed on each plasmid in the two strains, suggesting a potential plasmid mobilization ability. For the conjugation assays screened by colistin for the two strains, both recipients successfully captured two plasmids: one was the small *mcr-1-*carrying plasmid, and the other was the middle plasmid in each donor. Furthermore, for the E. coli EC15255, on the MH agar with the presence of ceftazidime (2 mg/L) and rifampicin (500 mg/L), a total of 16 colonies were selected, among which 8 colonies contained plasmid 2 and plasmid 3, other 8 colonies only contained plasmid 2. For the K. pneumoniae KP15255, on the MH agar with the presence of levofloxacin (2 mg/L) and rifampicin (500 mg/L), a total of 8 colonies were selected and all the 8 colonies only contained plasmid 2 (data not shown). The results indicated that the middle plasmid (plasmid 2) could transfer independently, and the small plasmid (plasmid 3) always transferred together with the middle plasmid. Hence, colistin resistance could be transferred to both recipients due to *mcr-1-*carrying plasmid mobilization, which should be assisted by the conjugable and middle plasmids they both harbored. So, we speculated that the middle plasmid might be a helper plasmid. The other antimicrobial resistances that were cotransferred could be explained by the relative resistance genes encoded by the middle plasmids, for instance, the *qnrS* gene in both strains for transferring quinolone resistance, the *bla*_CTX-M-14_ gene in pEC15255_2 for β-lactam resistance and the *aac(6′)-Ib-cr* gene in pKP15255_2 for aminoglycoside resistance. The conjugation ability should be the reason why the two strains shared a completely identical plasmid (the small one) and a very similar plasmid (the middle one), when they have a possible chance to stay together during the infection process in the same patient.

Evidence of plasmid plasticity has also been indicated by genetic events such as insertion, deletion, amplification, or inversion, most of which were observed in the E. coli and K. pneumoniae isolates we collected during the coinfection process. Comparison of the sequence of the middle-sized plasmid in each isolate revealed multiple events. Class II transposon Tn*As1* was confirmed to mediate *bla*_CTX-M-14_-carrying segment insertion, although in most previous observations, IS*Ecp1* was mainly responsible for *bla*_CTX-M-14_ gene transfer. Several genetic events were induced by the active mobile element IS*26*. IS*26* plays an important role in the genetic exchange between bacteria or plasmids, and extensively resistant Gram-negative bacteria often simultaneously carry several antimicrobial resistance genes and multiple copies of IS*26* (15). For instance, there are up to 10 copies of IS*26* located on the plasmid pKP15255_1. The transposition mechanism of IS*26* is generally regarded to involve replicative transposition and cointegrate formation (25–28), which is possibly responsible for mobilization of the *qnrS1*-carrying segment and the *bla*_CTX-M-14_ copy 1 segment located on plasmid pKP15255_1. Indeed, the high copy number observed in this study reflects the high activity of IS*26*, which is consistent with previous reports on other clinical *Enterobacteriales* isolates (29, 30).

Most of the *mcr-1*-encoding plasmids could be divided into three dominant Inc groups, IncX4, IncI2, IncHI2, and occasionally distributed in IncP1, p0111, etc. (31–33). In this study, both of the *mcr-1*-encoding plasmids from the E. coli and K. pneumoniae isolates belonged to the IncX4 type, which was conserved in the possibly plasmid exchange process (11, 34). These plasmids seemed relatively small and encoded almost no other resistance genes; even two copies of IS*Apl1* in the conserved Tn*6330*, which are commonly responsible for *mcr-1* transfer, were lacking, implying that the *mcr-1* gene has been fixed on this plasmid (11). Moreover, to the best of our knowledge, the spread of *mcr-1*-carrying plasmids between different species from the same infection site has not been reported.

As is well-known, the *mcr-1* gene encoded product MCR-1 usually mediated low level polymyxin resistance in Enterobacteriales, with the polymyxin MIC ranging from 4–8 mg/L mostly. But in K. pneumoniae and Enterobacter cloacae *complex*, the MCR-1 mediated polymyxin resistance is usually at a higher level. Our previous study reported 21 *mcr-1*-positive Enterobacteriaceae, and E. coli isolates carrying functional *mcr-1* gene showed polymyxin MICs ranged between 4 and 16 mg/L, while the polymyxin MIC of one K. pneumoniae carrying *mcr-1* gene was 32 mg/L (32). In addition, the polymyxin MIC value of *mcr-1*-positive Enterobacter cloacae *complex* could reach as high as >32 mg/L (35, 36). In the present study, the MIC of colistin for EC15255 is 4 mg/L, while for KP15255, the MIC is 16–32 mg/L. And when the *mcr-1*-carrying plasmid from KP15255 transferred to the E. coli EC600, the MIC of the transconjucant is 4 mg/L. We speculated that the expression of *mcr-1* gene or the copy numbers of *mcr-1*-carrying plasmid might be distinct in different bacterial species, but the definite mechanisms need to be investigated further.

In summary, the evidence in the interspecies communication of the plasmids proved that multiple genetic events, such as whole-plasmid transfer, insertion, deletion, amplification, or inversion, played a critical role in antimicrobial resistance gene transmission. Moreover, plasmid exchange poses a substantial threat due to the coexisting resistance genes in some plasmids and necessitates the continued surveillance of multidrug resistant pathogens.

## MATERIALS AND METHODS

### Bacterial isolates.

The two isolates were isolated from the abdominal drainage of a patient who suffered intestinal perforation and underwent surgical treatment; one was E. coli, and the other was K. pneumoniae, namely, isolates EC15255 and KP15255, respectively. The patient was admitted to a tertiary hospital from Zhejiang in September 2015. Both isolates showed colistin resistance by the Vitek 2 system (bioMérieux, France), and then they were stored then they were stored in Brain Heart Infusion (BHI) Broth with 20% glycerol at minus 80 degrees and further confirmed by matrix-assisted laser desorption ionization-time of flight mass spectrometry (MALDI-TOF) (Bruker Daltonics, Bremen, Germany).

### Antimicrobial susceptibility testing.

The MICs of several antimicrobial agents, including colistin, ceftazidime, cefepime, cefoperazone/sulbactam, piperacillin-tazobactam, ertapenem, meropenem, aztreonam, levofloxacin, amikacin, tigecycline, minocycline, tetracycline and sulfamethoxazole, for the two clinical isolates and their colistin-resistant transconjugants were determined by the broth microdilution method according to the Clinical and Laboratory Standards Institute (CLSI). All the MICs were performed three times. The results were interpreted in accordance with the CLSI (37) and European Committee on Antimicrobial Susceptibility Testing (EUCAST) (colistin and tigecycline) breakpoints (http://www.eucast.org/clinical_breakpoints).

### DNA extraction and genomic library preparation.

Genomic DNA was extracted for genome sequencing using the QIAamp DNA minikit (Qiagen, Germany). The DNA concentration was quantified using a NanoDrop 2000 (Thermo Scientific, USA) spectrophotometer and verified by agarose gel electrophoresis. An amount of extracted DNA > 50 ng was required for library preparation prior to whole-genome sequencing. Libraries were prepared using the TruePrepTM DNA Library Prep kit V2 for Illumina (Vazyme, China).

### Whole-genome sequencing (WGS) and analysis.

The whole genomes were sequenced by both the Illumina-HiSeq X 10 platform (Illumina Inc., USA) using a 150-bp paired-end reads protocol and the MinION platform (Nanopore, UK). The complete chromosome and plasmid sequences were obtained by hybrid assembling with Unicycler v.0.4.8 (38). Multilocus sequence typing (MLST), antimicrobial resistance genes and plasmid typing were performed using MLST 2.0, ResFinder 4.1 and PlasmidFinder 2.1, respectively, on the Center for Genomic Epidemiology server (http://genomicepidemiology.org/services/). Plasmid sequence annotation was performed with the RAST server (https://rast.nmpdr.org/). The IS and transposon elements were screened on ISFinder (https://www-is.biotoul.fr/). The mobile-associated genes of the plasmids were analyzed by the oriTFinder tool (https://tool-mml.sjtu.edu.cn/oriTfinder/oriTfinder.html). The sequence similarities among plasmids were visualized with Circos 07.09.16 (http://tools.bat.infspire.org/circoletto/) (39). Sequence comparisons were performed using BLAST (http://blast.ncbi.nlm.nih.gov) and sketched by Easyfig v.2.2.2.

### S1-PFGE, southern blot and conjugation assay.

To determine the location of the two *mcr-1* genes, genomic DNA digested with *S1* nuclease (TaKaRa, Japan) was electrophoresed on a CHEF-mapper XA pulsed-field gel electrophoresis (PFGE) system (Bio-Rad, USA) for 20 h at 14°C with run conditions of 6 V/cm and pulse times from 2.16 s to 63.8 s. The DNA fragments were transferred to a positively charged nylon membrane (Millipore, USA) and then hybridized with a digoxigenin-labeled *mcr-1*-specific probe. The fragments were then detected using an NBT/BCIP color detection kit (Roche, Germany). A filter mating test for conjugation was performed with both *mcr-1*-positive isolates using E. coli EC600 (rifampicin resistant) as the recipient strain. Transconjugants were selected on MH agar in the presence of colistin (2 mg/L) and rifampicin (500 mg/L) and further confirmed as *mcr-1*-positive by PCR analysis (40). The successful transconjugant of K. pneumoniae KP15255 was confirmed as E. coli by MODI-TOF and further confirmed as *mcr-1*-positive by PCR analysis. The successful transconjugant of E. coli EC15255 was confirmed by PFGE digested with XbaI-nuclease and further confirmed as *mcr-1*-positive by PCR analysis. Furthermore, the conjugation efficiencies were investigated the conjugation assays described above, and the transfer frequencies were calculated as the number of transconjugants obtained per recipient. All the experiments were performed for three biological repetitions and technical repetitions, respectively. The graph was drawn by GranphPad Prism and the difference of the conjugation efficiencies of the colistin resistance were analyzed by *unpaired t test*. *P* value was two-tailed, and a *p-v*alue of < 0.05 was considered statistically significant.

### Nucleotide sequence accession numbers.

The whole-genome sequences of E. coli EC15255 and K. pneumoniae KP15255 reported in this study have been deposited in the GenBank nucleotide database under BioProject PRJNA693056.

### Ethical approval.

The clinical isolates were part of the routine hospital laboratory procedure. The present study mainly focused on bacteria, but not the patient. Therefore, ethical approval was not needed.

## References

[1] Podschun R, Ullmann U. 1998. Klebsiella spp. as nosocomial pathogens: epidemiology, taxonomy, typing methods, and pathogenicity factors. Clin Microbiol Rev 11:589–603. doi:10.1128/CMR.11.4.589. 9767057 PMC88898

[2] Falagas ME, Karageorgopoulos DE, Nordmann P. 2011. Therapeutic options for infections with Enterobacteriaceae producing carbapenem-hydrolyzing enzymes. Future Microbiol 6:653–666. doi:10.2217/fmb.11.49. 21707312

[3] Biehl LM, Schmidt-Hieber M, Liss B, Cornely OA, Vehreschild MJ. 2016. Colonization and infection with extended spectrum beta-lactamase producing Enterobacteriaceae in high-risk patients - Review of the literature from a clinical perspective. Crit Rev Microbiol 42:1–16. doi:10.3109/1040841X.2013.875515. 24495097

[4] Canton R, Novais A, Valverde A, Machado E, Peixe L, Baquero F, Coque TM. 2008. Prevalence and spread of extended-spectrum beta-lactamase-producing Enterobacteriaceae in Europe. Clin Microbiol Infect 14 Suppl 1:144–153. doi:10.1111/j.1469-0691.2007.01850.x. 18154538

[5] De Oliveira DMP, Forde BM, Kidd TJ, Harris PNA, Schembri MA, Beatson SA, Paterson DL, Walker MJ. 2020. Antimicrobial resistance in ESKAPE pathogens. Clin Microbiol Rev 33:e00181-19. doi:10.1128/CMR.00181-19. 32404435 PMC7227449

[6] Robicsek A, Jacoby GA, Hooper DC. 2006. The worldwide emergence of plasmid-mediated quinolone resistance. Lancet Infect Dis 6:629–640. doi:10.1016/S1473-3099(06)70599-0. 17008172

[7] Strahilevitz J, Jacoby GA, Hooper DC, Robicsek A. 2009. Plasmid-mediated quinolone resistance: a multifaceted threat. Clin Microbiol Rev 22:664–689. doi:10.1128/CMR.00016-09. 19822894 PMC2772364

[8] Sun J, Zhang H, Liu YH, Feng Y. 2018. Towards understanding MCR-like colistin resistance. Trends Microbiol 26:794–808. doi:10.1016/j.tim.2018.02.006. 29525421

[9] Liu Y-Y, Wang Y, Walsh TR, Yi L-X, Zhang R, Spencer J, Doi Y, Tian G, Dong B, Huang X, Yu L-F, Gu D, Ren H, Chen X, Lv L, He D, Zhou H, Liang Z, Liu J-H, Shen J. 2016. Emergence of plasmid-mediated colistin resistance mechanism MCR-1 in animals and human beings in China: a microbiological and molecular biological study. Lancet Infect Dis 16:161–168. doi:10.1016/S1473-3099(15)00424-7. 26603172

[10] Norman A, Hansen LH, Sorensen SJ. 2009. Conjugative plasmids: vessels of the communal gene pool. Philos Trans R Soc Lond B Biol Sci 364:2275–2289. doi:10.1098/rstb.2009.0037. 19571247 PMC2873005

[11] Jiang Y, Zhang Y, Lu J, Wang Q, Cui Y, Wang Y, Quan J, Zhao D, Du X, Liu H, Li X, Wu X, Hua X, Feng Y, Yu Y. 2020. Clinical relevance and plasmid dynamics of mcr-1-positive Escherichia coli in China: a multicentre case-control and molecular epidemiological study. Lancet Microbe 1:e24–e33. doi:10.1016/S2666-5247(20)30001-X. 35538906

[12] Carattoli A. 2009. Resistance plasmid families in Enterobacteriaceae. Antimicrob Agents Chemother 53:2227–2238. doi:10.1128/AAC.01707-08. 19307361 PMC2687249

[13] Rozwandowicz M, Brouwer MSM, Fischer J, Wagenaar JA, Gonzalez-Zorn B, Guerra B, Mevius DJ, Hordijk J. 2018. Plasmids carrying antimicrobial resistance genes in Enterobacteriaceae. J Antimicrob Chemother 73:1121–1137. doi:10.1093/jac/dkx488. 29370371

[14] Wiedenbeck J, Cohan FM. 2011. Origins of bacterial diversity through horizontal genetic transfer and adaptation to new ecological niches. FEMS Microbiol Rev 35:957–976. doi:10.1111/j.1574-6976.2011.00292.x. 21711367

[15] Partridge SR, Kwong SM, Firth N, Jensen SO. 2018. Mobile genetic elements associated with antimicrobial resistance. Clin Microbiol Rev 31:e00088-17. doi:10.1128/CMR.00088-17. 30068738 PMC6148190

[16] Chaisson MJ, Pevzner PA. 2008. Short read fragment assembly of bacterial genomes. Genome Res 18:324–330. doi:10.1101/gr.7088808. 18083777 PMC2203630

[17] Conlan S, Thomas PJ, Deming C, Park M, Lau AF, Dekker JP, Snitkin ES, Clark TA, Luong K, Song Y, Tsai YC, Boitano M, Dayal J, Brooks SY, Schmidt B, Young AC, Thomas JW, Bouffard GG, Blakesley RW, Program NCS, Mullikin JC, Korlach J, Henderson DK, Frank KM, Palmore TN, Segre JA, NISC Comparative Sequencing Program. 2014. Single-molecule sequencing to track plasmid diversity of hospital-associated carbapenemase-producing Enterobacteriaceae. Sci Transl Med 6:254ra126. doi:10.1126/scitranslmed.3009845. PMC4203314 25232178

[18] Gorrie CL, Mirceta M, Wick RR, Judd LM, Lam MMC, Gomi R, Abbott IJ, Thomson NR, Strugnell RA, Pratt NF, Garlick JS, Watson KM, Hunter PC, Pilcher DV, McGloughlin SA, Spelman DW, Wyres KL, Jenney AWJ, Holt KE. 2022. Genomic dissection of Klebsiella pneumoniae infections in hospital patients reveals insights into an opportunistic pathogen. Nat Commun 13:3017. doi:10.1038/s41467-022-30717-6. 35641522 PMC9156735

[19] Halary S, Leigh JW, Cheaib B, Lopez P, Bapteste E. 2010. Network analyses structure genetic diversity in independent genetic worlds. Proc Natl Acad Sci USA 107:127–132. doi:10.1073/pnas.0908978107. 20007769 PMC2806761

[20] Mohamed NM, Zakaria AS, Edward EA. 2022. Genomic characterization of international high-risk clone ST410 Escherichia coli co-harboring ESBL-encoding genes and bla(NDM-5) on IncFIA/IncFIB/IncFII/IncQ1 multireplicon plasmid and carrying a chromosome-borne bla(CMY-2) from Egypt. Antibiotics (Basel) 11:1031. doi:10.3390/antibiotics11081031. 36009900 PMC9405272

[21] Pankok F, Taudien S, Dekker D, Thye T, Oppong K, Wiafe Akenten C, Lamshoft M, Jaeger A, Kaase M, Scheithauer S, Tanida K, Frickmann H, May J, Loderstadt U. 2022. Epidemiology of plasmids in Escherichia coli and Klebsiella pneumoniae with acquired extended spectrum Beta-lactamase genes isolated from chronic wounds in Ghana. Antibiotics (Basel) 11:689–702. doi:10.3390/antibiotics11050689. 35625333 PMC9138140

[22] Zhao Q, Feng Y, Zong Z. 2022. An integrated IncFIB/IncFII plasmid confers hypervirulence and its fitness cost and stability. Eur J Clin Microbiol Infect Dis 41:681–684. doi:10.1007/s10096-022-04407-6. 35044544

[23] Rooney CM, Sheppard AE, Clark E, Davies K, Hubbard ATM, Sebra R, Crook DW, Walker AS, Wilcox MH, Chilton CH. 2019. Dissemination of multiple carbapenem resistance genes in an in vitro gut model simulating the human colon. J Antimicrob Chemother 74:1876–1883. doi:10.1093/jac/dkz106. 30989197

[24] Rodríguez-Santiago J, Cornejo-Juárez P, Silva-Sánchez J, Garza-Ramos U. 2021. Polymyxin resistance in Enterobacterales: overview and epidemiology in the Americas. Int J Antimicrob Agents 58:106426. doi:10.1016/j.ijantimicag.2021.106426. 34419579

[25] Harmer CJ, Hall RM. 2020. IS26 family members IS257 and IS1216 also form cointegrates by copy-in and targeted conservative routes. mSphere 5:e00811-19. doi:10.1128/mSphere.00811-19. 31915227 PMC6952201

[26] Harmer CJ, Hall RM. 2016. IS26-mediated formation of transposons carrying antibiotic resistance genes. mSphere 1:e00038-16. doi:10.1128/mSphere.00038-16. PMC4894685 27303727

[27] Harmer CJ, Hall RM. 2015. IS26-mediated precise excision of the IS26-aphA1a translocatable unit. mBio 6:e01866-15–e01815. doi:10.1128/mBio.01866-15. 26646012 PMC4676283

[28] Harmer CJ, Moran RA, Hall RM. 2014. Movement of IS26-associated antibiotic resistance genes occurs via a translocatable unit that includes a single IS26 and preferentially inserts adjacent to another IS26. mBio 5:e01801-14–e01814. doi:10.1128/mBio.01801-14. 25293759 PMC4196232

[29] Miriagou V, Carattoli A, Tzelepi E, Villa L, Tzouvelekis LS. 2005. IS26-associated In4-type integrons forming multiresistance loci in enterobacterial plasmids. Antimicrob Agents Chemother 49:3541–3543. doi:10.1128/AAC.49.8.3541-3543.2005. 16048979 PMC1196216

[30] Lee SY, Park YJ, Yu JK, Jung S, Kim Y, Jeong SH, Arakawa Y. 2012. Prevalence of acquired fosfomycin resistance among extended-spectrum beta-lactamase-producing Escherichia coli and Klebsiella pneumoniae clinical isolates in Korea and IS26-composite transposon surrounding fosA3. J Antimicrob Chemother 67:2843–2847. doi:10.1093/jac/dks319. 22893681

[31] Zhong L-L, Phan HTT, Shen C, Vihta K-D, Sheppard AE, Huang X, Zeng K-J, Li H-Y, Zhang X-F, Patil S, Crook DW, Walker AS, Xing Y, Lin J-L, Feng L-Q, Doi Y, Xia Y, Stoesser N, Tian G-B. 2018. High rates of human fecal carriage of mcr-1-positive multidrug-resistant Enterobacteriaceae Emerge in China in association with successful plasmid families. Clin Infect Dis 66:676–685. doi:10.1093/cid/cix885. 29040419 PMC5848316

[32] Quan J, Li X, Chen Y, Jiang Y, Zhou Z, Zhang H, Sun L, Ruan Z, Feng Y, Akova M, Yu Y. 2017. Prevalence of mcr-1 in Escherichia coli and Klebsiella pneumoniae recovered from bloodstream infections in China: a multicentre longitudinal study. Lancet Infect Dis 17:400–410. doi:10.1016/S1473-3099(16)30528-X. 28139430

[33] Li B, Ke B, Zhao X, Guo Y, Wang W, Wang X, Zhu H. 2018. Antimicrobial resistance profile of mcr-1 positive clinical isolates of Escherichia coli in China from 2013 to 2016. Front Microbiol 9:2514. doi:10.3389/fmicb.2018.02514. 30405572 PMC6206212

[34] Gao R, Hu Y, Li Z, Sun J, Wang Q, Lin J, Ye H, Liu F, Srinivas S, Li D, Zhu B, Liu YH, Tian GB, Feng Y. 2016. Dissemination and mechanism for the MCR-1 colistin resistance. PLoS Pathog 12:e1005957. doi:10.1371/journal.ppat.1005957. 27893854 PMC5125707

[35] Wong SC, Tse H, Chen JH, Cheng VC, Ho PL, Yuen KY. 2016. Colistin-resistant Enterobacteriaceae carrying the mcr-1 Gene among patients in Hong Kong. Emerg Infect Dis 22:1667–1669. doi:10.3201/eid2209.160091. 27532341 PMC4994376

[36] Zeng K-J, Doi Y, Patil S, Huang X, Tian G-B. 2016. Emergence of the plasmid-mediated mcr-1 gene in colistin-resistant Enterobacter aerogenes and Enterobacter cloacae. Antimicrob Agents Chemother 60:3862–3863. doi:10.1128/AAC.00345-16. 26976876 PMC4879368

[37] Anonymous. CLSI. Performance Standards for Antimicrobial Susceptibility Testing. 31st ed CLSI supplement M100. Clinical and Laboratory Standards Institute; 2021. 10.1128/JCM.00213-21 PMC8601225 34550809

[38] Wick RR, Judd LM, Gorrie CL, Holt KE. 2017. Unicycler: Resolving bacterial genome assemblies from short and long sequencing reads. PLoS Comput Biol 13:e1005595. doi:10.1371/journal.pcbi.1005595. 28594827 PMC5481147

[39] Krzywinski M, Schein J, Birol I, Connors J, Gascoyne R, Horsman D, Jones SJ, Marra MA. 2009. Circos: an information aesthetic for comparative genomics. Genome Res 19:1639–1645. doi:10.1101/gr.092759.109. 19541911 PMC2752132

[40] Li X, Mu X, Zhang P, Zhao D, Ji J, Quan J, Zhu Y, Yu Y. 2018. Detection and characterization of a clinical Escherichia coli ST3204 strain coproducing NDM-16 and MCR-1. Infect Drug Resist 11:1189–1195. doi:10.2147/IDR.S175041. 30147347 PMC6101002

