# Supplementary material for: Investigating Possible Interspecies Communication of Plasmids Associated with Transfer of Third-Generation Cephalosporin, Quinolone, and Colistin Resistance Between Simultaneously Isolated Escherichia Coli and Klebsiella Pneumoniae
Source: Microbiol Spectr. 2023 May 1;11(3):e03554-22. doi: 10.1128/spectrum.03554-22 (PMC10269620; doi:10.1128/spectrum.03554-22)
Supplement: Supplemental file 1 — Table S1 and Fig. S1. Download spectrum.03554-22-s0001.pdf, PDF file, 0.2 MB [file spectrum.03554-22-s0001.pdf]

Supplementary table 1. Mobile associated genes of the plasmids of EC15255 and KP15255

| Isolates | Plasmid 1 | Plasmid 2 | Plasmid 3 |
|---|---|---|---|
| EC15255 | *traA, traB, traC, traD, traE, traF, traG, traH, traK, traL, traM, traN, traQ, traU, traV, traW, traX, traY, trbC* | *trwD, trwE, trwF, trwG, trwH, trw I, trwJ, trwK, trwL, trwM, trwN, magB05* | *virB1, virB2, virB4, virB5, virB6, virB8, virB9, virB10, virB11, virD4, magB05* |
| KP15255 | *traA, traB, traC, traD, traE, traF, traG, traH, traK, traL, traM, traN, traQ, traU, traV, traW, traX, trbC* | *trwD, trwE, trwF, trwG, trw I, trwJ, trwK, trwL, trwM, trwN, magB05* | *virB1, virB2, virB4, virB5, virB6, virB8, virB9, virB10, virB11, virD4, magB05* |

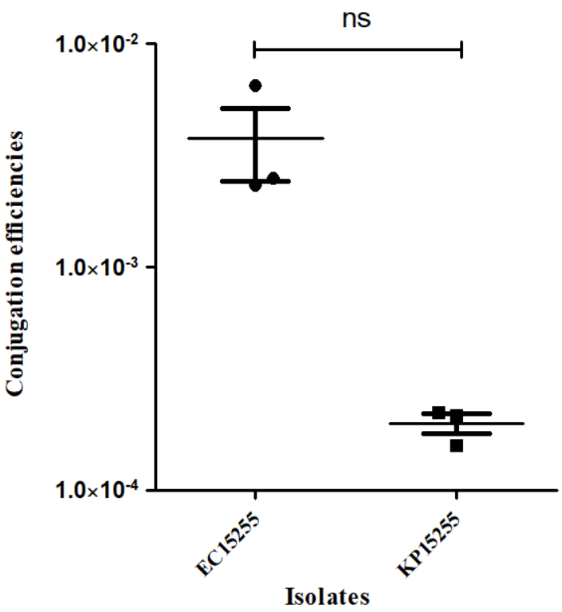


Supplementary figure 1. The conjugation efficiencies of colistin resistance from isolates EC15255 and KP15255

ns: no statistic difference ($p$=0.0582)
